# Large-scale Lassa fever outbreaks in Nigeria: quantifying the association between disease reproduction number and local rainfall

**DOI:** 10.1017/S0950268819002267

**Published:** 2020-01-10

**Authors:** Shi Zhao, Salihu S. Musa, Hao Fu, Daihai He, Jing Qin

**Affiliations:** 1 School of Nursing, Hong Kong Polytechnic University, Hong Kong, China; 2 Department of Applied Mathematics, Hong Kong Polytechnic University, Hong Kong, China; 3 Division of Biostatistics, JC School of Public Health and Primary Care, Chinese University of Hong Kong, Hong Kong, China; 4 Clinical Trials and Biostatistics Lab, Shenzhen Research Institute, Chinese University of Hong Kong, Shenzhen, China; 5 Department of Crop Science and Technology, College of Agriculture, South China Agricultural University, Guangzhou, China

**Keywords:** Lassa fever, modelling analysis, Nigeria, rainfall, reproduction number, spatial heterogeneity

## Abstract

Lassa fever (LF) is increasingly recognised as an important rodent-borne viral haemorrhagic fever presenting a severe public health threat to sub-Saharan West Africa. In 2017–18, LF caused an unprecedented epidemic in Nigeria and the situation was worsening in 2018–19. This work aims to study the epidemiological features of epidemics in different Nigerian regions and quantify the association between reproduction number (*R*) and state rainfall. We quantify the infectivity of LF by the reproduction numbers estimated from four different growth models: the Richards, three-parameter logistic, Gompertz and Weibull growth models. LF surveillance data are used to fit the growth models and estimate the *R*s and epidemic turning points (*τ*) in different regions at different time periods. Cochran's *Q* test is further applied to test the spatial heterogeneity of the LF epidemics. A linear random-effect regression model is adopted to quantify the association between *R* and state rainfall with various lag terms. Our estimated *R*s for 2017–18 (1.33 with 95% CI 1.29–1.37) was significantly higher than those for 2016–17 (1.23 with 95% CI: (1.22, 1.24)) and 2018–19 (ranged from 1.08 to 1.36). We report spatial heterogeneity in the *R*s for epidemics in different Nigerian regions. We find that a one-unit (mm) increase in average monthly rainfall over the past 7 months could cause a 0.62% (95% CI 0.20%–1.05%)) rise in *R*. There is significant spatial heterogeneity in the LF epidemics in different Nigerian regions. We report clear evidence of rainfall impacts on LF epidemics in Nigeria and quantify the impact.

## Introduction

Lassa fever (LF), caused by Lassa virus (LASV), is increasingly recognised as an important rodent-borne viral haemorrhagic fever presenting a severe public health threat to some of the communities in sub-Saharan West Africa [1]. Discovered in 1969 [2], LF is endemic to much of rural Nigeria and regions in the Mano River Union [3]. LASV transmits from human to human, as well as via the zoonotic cycle [1, 3, 4]. LF has a high case fatality rate ranging from 1% in the community to over 60% in hospital settings [1, 4, 5]. The common reservoir of LASV is *Mastomys natalensis*, one of the most widespread rodent species in sub-Saharan Africa [1, 3], which exhibits sensitive population dynamics to the water level, e.g. rainfall, flooded agricultural activities [6, 7]. Previous studies have recognised the ecological association between the population levels of rodents and rainfall [8–10].

LF epidemics typically start in November and last until May of the following year, with the majority of cases occurring in the first quarter of the following year, in addition to sporadic cases reported throughout the year. The 2017–18 epidemic in Nigeria was an unprecedented LF epidemic in the country's history [11], which resulted in 400 confirmed cases, including 97 deaths, between January and March 2018 [12]. The most recent epidemic in Nigeria has already caused 526 confirmed cases from January to March of 2019, which included 121 deaths [12]. The five states of Edo, Ondo, Ebonyi, Bauchi and Plateau are the only states that have been among the top 10 hit hardest states in terms of number of LF cases in both the 2018 (85.5% of total national cases) and 2019 (85.7% of total national cases) epidemics. While there have been discussions about the association of rainfall level and LF incidence rate [13, 14], this association has not yet been demonstrated and quantified. This work aims to study the epidemiological features of epidemics in different Nigerian regions between January 2016 and March 2019. We estimate LF infectivity in terms of the reproduction number (*R*) and quantify the association between *R* and state rainfall. We explore the spatial heterogeneity of the LF epidemics and summarise the overall findings with model-average estimates.

## Data and methods

### Data

**Fig. 1.**
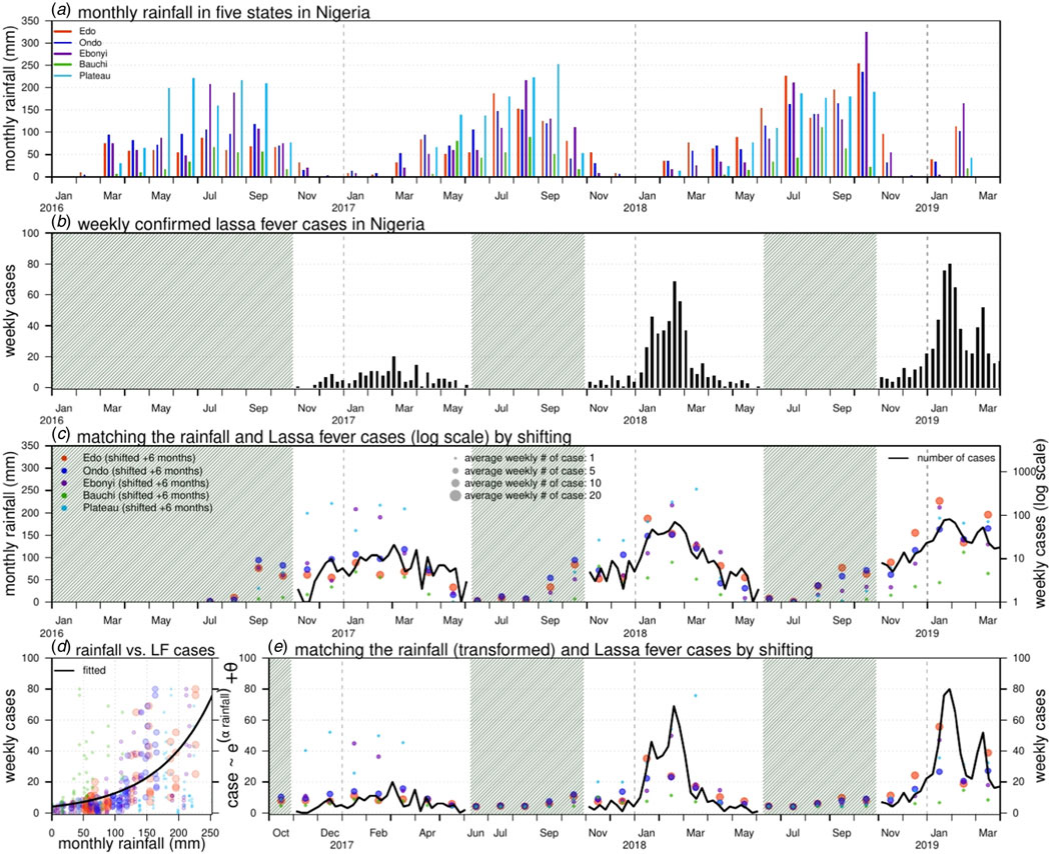
Rainfall (unit: mm) and number of Lassa fever (LF) cases in Nigeria. Panel (a) shows the monthly rainfall in five states in Nigeria. Panel (b) shows the weekly number of LF cases in Nigeria. The shaded area represents a weekly number of cases lower than 10. Panel (c) matches the rainfall (dots) and LF cases (in log scale, black line) by shifting the rainfall time series by + 6 months. The sizes of each dot represent the number of the average weekly LF cases in each state in the 2017–18 and 2018–19 outbreaks. Panel (d) is the scatter plot of rainfall (shifted + 6 months) *vs.* LF cases; the dots of different colours and sizes share the same scheme as in panel (c). The black line is the fitting outcome of the formula ‘case ~ exp(*α* × rainfall) + *θ*’ by least square estimation and here, the ‘rainfall’ is the rainfall time series shifted + 6 months. The fitted *R*-squared is 0.41 and significance is *P*-value < 0.0001. Panel (e) is the fitting outcome from panel (d) and the rainfall dots (shifted + 6 months) of different colours and sizes share the same scheme as in panel (c).

Weekly LF surveillance data are obtained from the Nigeria Centre for Disease Control (NCDC), where the data are publicly available from the weekly situation reports released by NCDC [12]. Laboratory-confirmed case time series are used for analysis. We examine the major epidemics that occurred between January 2016 and March 2019 across the whole country and the aforementioned five states that were among the top 10 hardest-hit states in both the 2018 and 2019 epidemics, i.e. Edo, Ondo, Ebonyi, Bauchi and Plateau. The state rainfall records of each state were collected on monthly average basis from the historical records of the World Weather Online website [15]. Figure 1(a) and (b) shows the rainfall time series of the five states and the weekly reported LF cases across the entire Nigeria.

### Intuitive coincidence between rainfall and epidemic

To test the credibility of the coincidence between rainfall and LF epidemic, we use a simple statistical regression model of ‘case ~ exp(*α* × rainfall) + *θ*', where *α* and *θ* are free parameters to be estimated. The ‘rainfall’ in the model represents the state rainfall time series with lag of 4–9 months. This lag term corresponds to the time interval between the rainfall and the development of rodent population [7]. We check the least-square fitting outcomes of these regression models and select the model of lagged rainfall with the highest goodness-of-fit. The fitting significance is treated as the initiation of the quantitative association between state rainfall and the LF epidemic.

### Modelling and estimation

**Fig. 2.**
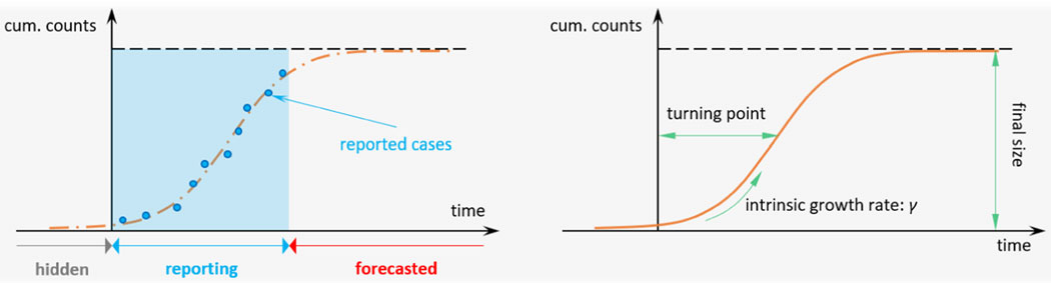
The illustration diagram of the growth models fitting framework. The (solid and dashed) orange lines are the theoretical growth curves from the simple nonlinear growth models, i.e. the Richards, logistic, Gompertz, or Weibull models. The blue dots are the reported cumulative (cum.) number of cases. The blue shading area represents the period with epidemic reported, which is used for the model fitting in corresponds to the non-shaded area in Figure 1. The intrinsic growth rate is the *γ* in Eqn (1), which is estimated from the fitted growth models and used for *R* estimation.

Four different nonlinear growth models are adopted to pinpoint the epidemiological features of each epidemic. The models are the Richards, three-parameter logistic, Gompertz and Weibull growth models. These simple structured models are widely used to study S-shaped cumulative growth processes; e.g. the curve of a single-wave epidemic and have been extensively studied in previous work [16, 17]. These models consider cumulative cases with saturation in the growth rate to reflect the progression of an epidemic due to reduction in susceptible pools or a decrease in the exposure to infectious rodent populations. The extrinsic growth rate increases to a maximum (i.e. saturation) before steadily declining to zero. The modelling and fitting via the growth models of the epidemic curve are illustrated in Figure 2.

We fit all models to the weekly reported LF cases in different regions and evaluate the fitting performance by the Akaike information criterion (AIC). We adopt the standard nonlinear least squares (NLS) approach for model fitting and parameter estimation, following [16, 18]. A *P*-value <0.05 is regarded as statistically significant and the 95% confidence intervals (CIs) are estimated for all unknown parameters. As we are using the cumulative number of the LF cases to conduct the model fitting, some fitting issues might occur, as per the studies in King *et al*. [19], due to the non-decreasing nature in the cumulative summation time series. The models are selected by comparing the AIC to that of the baseline (or null) model. Only the models with an AIC lower than the AIC of the baseline model are considered for further analysis. Importantly, the baseline model adopted is expected to capture the trends of the time series. Since the epidemic curves of an infectious disease commonly exhibit autocorrelations [20], we use autoregression (AR) models with a degree of 2, i.e. AR(2), as the baseline models for growth model selection. We also adopt the coefficient of determination (*R*-squared) and the coefficient of partial determination (partial *R*-squared) to evaluate goodness-of-fit and fitting improvement, respectively. For the calculation of partial *R*-squared, the AR(2) model is used as the baseline model. The growth models with a positive partial *R*-squared (indicating fitting improvement) against the baseline AR(2) model will be selected for further analyses.

After the selection of models, we estimate the epidemiological features (parameters) of turning point (*τ*) and reproduction number (*R*) via the selected models. The turning point is defined as the time point of a sign change in the rate of case accumulation, i.e. from increasing to decreasing or *vice versa* [16, 18]. The reproduction number, *R*, is the average number of secondary human cases caused by one primary human case via the ‘human-to-rodent-to-human’ transmission path [18, 21]. When the population is totally (i.e. 100%) susceptible, the *R* will equate to the basic reproduction number, commonly denoted as *R*_0_ [21, 22]. The reproduction number (*R*) is given in Eqn (1),
1
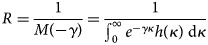
Here, *γ* is the intrinsic per capita growth rate from the nonlinear growth models and *κ* is the serial interval of the LASV infection. The serial interval (i.e. the generation interval) is the time between the infections of two successive cases in a chain of transmission [21, 23–25]. The function *h*(·) represents the probability distribution of *κ*. Hence, the function *M*(·) is the Laplace transform of *h*(·) and specifically, *M*(·) is the moment generating function (MGF) of a probability distribution [21]. According to previous work [26], we assume *h*(*κ*) to follow a Gamma distribution with a mean of 7.8 days and a standard deviation (SD) of 10.7 days. Therefore, *R* can be estimated with the values of *γ* from the fitted models [18, 21, 27, 28]. The state *R*s were estimated from the *γ*s of the fitted epidemic growth curves of each state. Similarly, the national *R*s are estimated from the *γ*s of the epidemic growth curves fitted to the national number of cases time series in different epidemic periods.

We then summarise the *κ* and *R* estimates via the AIC-weighted model averaging. The AIC weights, *w*, of the selected models (with positive partial *R*-squared) are defined in Eqn (2),
2
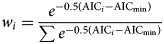
Here, AIC_*i*_ is the AIC of the *i*-th selected model and the AIC_min_ is the lowest AIC among all selected models. Thus, the *i*-th selected model has a weight of *w*_*i*_. The model-averaged estimator is the weighted average of the estimates in each selected model, which has been well studied in previous work [16, 29].

For the AIC-based model average of the *R*, there could be the situation that no growth model is selected according to the partial *R*-squared. In such cases, instead of the model average, we report the range of the *R* estimated from all growth models.

### Testing the spatial heterogeneity of the LF epidemics

After finding the model-averaged estimates, we apply Cochran's *Q* test to examine the spatial heterogeneity of the epidemics in different regions over the same period of time [30]. For instance, we treat the model-averaged *R* estimates as the univariate meta-analytical response against different Nigerian regions (states) and further check the heterogeneity by estimating the significance levels of the *Q* statistics. A *P*-value <0.05 is regarded as statistically significant.

### Association between rainfall and reproduction number

**Fig. 3.**
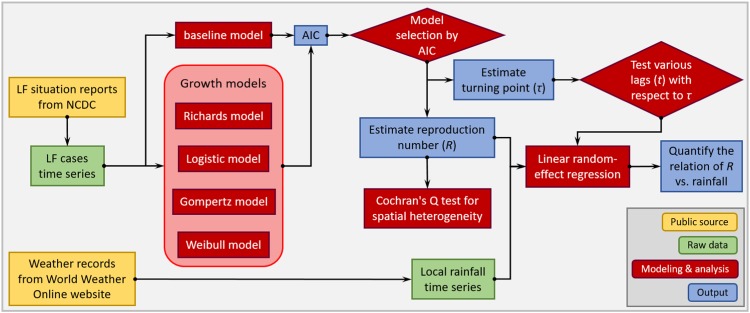
A flow diagram of the modelling analysis. This figure shows the analysis procedures in this study.

Similar to the approach in the previous study [31], the association between the state rainfall level and LASV transmissibility are modelled by a linear mixed-effect regression (LMER) model in Eqn (3),
3
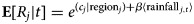
Here, **E**(·) represents the expectation function and *j* is the region index corresponding to different regions (states). Term *c*_*j*_ is the interception term of the *j*-th region to be estimated and it is variable from different regions, serving as the baseline scale of transmissibility in different states. The term *t* denotes the cumulative lag in the model and 〈rainfall_*j,t*_〉 represents the average monthly rainfall of the previous *t* months from the turning point, *τ*, of the *j*-th region. The range of lag term, *t*, will be considered from 4 to 9 months, which is explained by the time interval between the peak of the rainfall and the peak of rodent population [7]. As illustrated in Figure 3, the reproduction numbers, *R*_*j*_s, are estimated for different epidemics from the selected growth models. The regression coefficient, *β*, is to be estimated. Hence, the term (*e^β^* – 1) × 100% is the percentage changing rate (of *R*), which can be interpreted as the percentage change in transmissibility due to a one-unit (mm) increase in the average of the monthly rainfall level over the past 7 months. The framework of the regression is based on the exponential form of the predictor to model the expectation of transmissibility (e.g. *R*); this framework is inspired by previous work [32–35]. To quantify the impacts of state rainfall, we calculate the percentage changing rate with different cumulative lags (*t*) from 4 to 9 months and estimate their significant levels. Only the lag terms (*t*) with significant estimates are presented in this work.

We present the analysis procedure in a flow diagram in Figure 3. All analyses are conducted by using **R** (version 3.4.3 [36]) and the **R** function ‘nls' is employed for the NLS estimation of model parameters.

## Results and discussion

The rainfall time series of the five states and the weekly reported LF cases of the whole of Nigeria are shown in Figure 1(a) and (b). We observe that the major LF epidemics usually occur in Nigeria between November and May of the following year. The cumulative lagged effects were observed via matching the peak timing of the rainfall and epidemic curves. In Figure 1(c), we shift the rainfall time series of the five states by + 6 months to match the trends of the national LF epidemic curve in Nigeria. In Figure 1(d) and (e), we find that the fit has a *P*-value <0.0001, which indicates a statistically significant association between the LF cases and shifted rainfall curve.

**Fig. 4.**
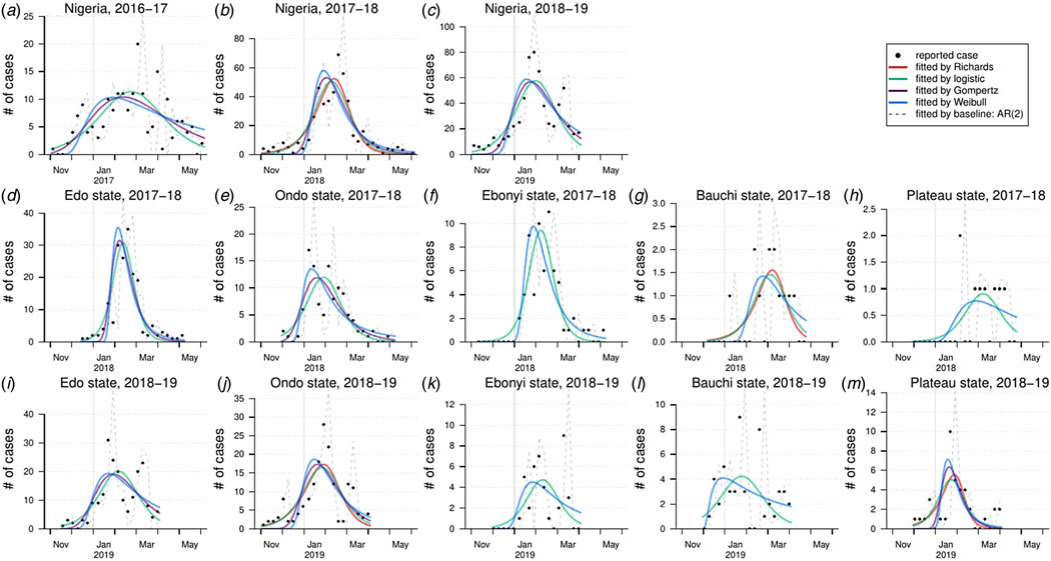
The fitting results of estimates of Lassa epidemics in Nigeria by nonlinear growth models. In each panel, the dots are the observed (reported) cases, the dashed grey line is the fit by the baseline AR(2) model and the coloured lines are the fits from the nonlinear growth models.

We fit four different growth models to the LF confirmed cases and estimate the model-average reproduction number (*R*) after model selection. We show the growth model fitting results in Figure 4 and the model estimation and selection results in Table 1. Most of the models have positive partial *R*-squared against the baseline AR(2) model. Most of the regions exhibit an epidemic turning point (*τ*) ranging from the epidemiological week (EW) 4–10, i.e. from the end of January to mid-March, in each year. Out of four epidemics in the states of Bauchi and Plateau, there are three estimated *τ*s after EW 10 (Table 1). Larger *τ* means the more extension in the duration of the epidemics. The turning point (*τ*) could be affected by several factors including seasonality, intervention program and depletion of the susceptible pool. The estimated reproduction number (*R*) of the epidemics in different regions varies from 1.06 to 1.62. At the national level, the *R* value for the whole of Nigeria in 2016–17 (*R* = 1.23 with 95% CI 1.22–1.24) is significantly lower than in the epidemics of 2017–18 (*R* = 1.33 with 95% CI 1.29–1.37) and 2018–19 (*R* ranged from 1.08 to 1.36). The state of Edo has the highest estimated *R* (1.62 in 2017–18 and 1.09 in 2018–19) and this state also has the largest number of LF cases in the epidemics of both 2017–18 (41.9% of all cases) and 2018–19 (36.0% of all cases). Hypothesised spatial heterogeneity in the *R* is tested via Cochran's *Q* test. The testing results for the *R*s in the five states are significant (i.e. *P*-value <0.05) for both the 2017–18 and 2018–19 LF epidemics. Thus, we report the existence of spatial heterogeneity in LF epidemics in Nigeria.

**Table 1.** The summary table of the model estimations. Population numbers are summarised in units of one million

Region	Population	Model	Epidemic period	Final size	Reproduction number	Turning point	*R*-squared	Improvement	AIC	Weight
Nigeria	186	Baseline: AR(2)	from EW44-2016 to EW22-2017	NA	NA	NA	0.9959	0.00%	198.8	NA
Nigeria	186	Richards	from EW44-2016 to EW22-2017	–	–	–	–	–	–	–
Nigeria	186	Logistic	from EW44-2016 to EW22-2017	196; (191, 201)	1.24; (1.22, 1.25)	EW10-2017; (EW10-2017, EW10-2017)	0.9973	34.80%	191.8	0.968
Nigeria	186	Gompertz	from EW44-2016 to EW22-2017	221; (208, 233)	1.14; (1.12, 1.15)	EW08-2017; (EW08-2017, EW09-2017)	0.9969	23.90%	198.6	0.032
Nigeria	186	Weibull	from EW44-2016 to EW22-2017	350; (252, 448)	1.05; (1.04, 1.07)	EW14-2017; (EW09-2017, EW18-2017)	0.9937	−53.80%	220.5	excluded
Nigeria	186	Model average	from EW44-2016 to EW22-2017	197; (192, 202)	1.23; (1.22, 1.24)	EW10-2017; (EW10-2017, EW10-2017)	NA	NA	NA	NA
Nigeria	190.9	Baseline: AR(2)	from EW44-2017 to EW22-2018	NA	NA	NA	0.9977	0.00%	157	NA
Nigeria	190.9	Richards	from EW44-2017 to EW22-2018	464; (458, 470)	1.33; (1.28, 1.37)	EW09-2018; (EW08-2018, EW09-2018)	0.9984	32.00%	151.2	0.933
Nigeria	190.9	Logistic	from EW44-2017 to EW22-2018	468; (462, 474)	1.41; (1.39, 1.43)	EW08-2018; (EW08-2018, EW08-2018)	0.998	14.00%	156.5	0.067
Nigeria	190.9	Gompertz	from EW44-2017 to EW22-2018	474; (465, 483)	1.31; (1.29, 1.34)	EW07-2018; (EW07-2018, EW07-2018)	0.9972	−20.60%	169	excluded
Nigeria	190.9	Weibull	from EW44-2017 to EW22-2018	491; (475, 507)	1.08; (1.08, 1.08)	EW07-2018; (EW07-2018, EW07-2018)	0.9955	−91.10%	183.3	excluded
Nigeria	190.9	Model average	from EW44-2017 to EW22-2018	464; (458, 470)	1.33; (1.29, 1.37)	EW09-2018; (EW08-2018, EW09-2018)	NA	NA	NA	NA
Nigeria	195.9	Baseline: AR(2)	from EW44-2018 to EW13-2019	NA	NA	NA	0.9969	0.00%	98.8	NA
Nigeria	195.9	Richards	from EW44-2018 to EW13-2019	–	–	–	–	–	–	–
Nigeria	195.9	Logistic	from EW44-2018 to EW13-2019	623; (593, 653)	1.36; (1.32, 1.39)	EW06-2019; (EW06-2019, EW07-2019)	0.9953	−54.00%	111.5	excluded
Nigeria	195.9	Gompertz	from EW44-2018 to EW13-2019	669; (620, 717)	1.24; (1.21, 1.28)	EW05-2019; (EW05-2019, EW06-2019)	0.9957	−39.40%	111.3	excluded
Nigeria	195.9	Weibull	from EW44-2018 to EW13-2019	795; (670, 921)	1.08; (1.08, 1.09)	EW06-2019; (EW05-2019, EW07-2019)	0.9951	−0.599	114.3	excluded
Nigeria	195.9	Model average	from EW44-2018 to EW13-2019	NA	NA	NA	NA	NA	NA	NA
Edo state	4.2	Baseline: AR(2)	from EW50-2017 to EW18-2018	NA	NA	NA	0.9901	0.00%	127.7	NA
Edo state	4.2	Richards	from EW50-2017 to EW18-2018	–	–	–	–	–	–	–
Edo state	4.2	Logistic	from EW50-2017 to EW18-2018	175; (173, 178)	1.62; (1.58, 1.66)	EW09-2018; (EW08-2018, EW09-2018)	0.9984	83.70%	95.6	0.995
Edo state	4.2	Gompertz	from EW50-2017 to EW18-2018	178; (174, 181)	1.46; (1.42, 1.50)	EW08-2018; (EW08-2018, EW08-2018)	0.9976	75.40%	106.2	0.005
Edo state	4.2	Weibull	from EW50-2017 to EW18-2018	182; (176, 189)	1.13; (1.13, 1.13)	EW08-2018; (EW08-2018, EW08-2018)	0.9963	62.20%	115.3	0
Edo state	4.2	Model average	from EW50-2017 to EW18-2018	175; (173, 178)	1.62; (1.58, 1.66)	EW09-2018; (EW08-2018, EW09-2018)	NA	NA	NA	NA
Ondo state	4.7	Baseline: AR(2)	from EW48-2017 to EW18-2018	NA	NA	NA	0.9917	0.00%	129.2	NA
Ondo state	4.7	Richards	from EW48-2017 to EW18-2018	–	–	–	–	–	–	–
Ondo state	4.7	Logistic	from EW48-2017 to EW18-2018	111; (108, 113)	1.41; (1.37, 1.45)	EW07-2018; (EW06-2018, EW07-2018)	0.9946	35.40%	124.6	0.007
Ondo state	4.7	Gompertz	from EW48-2017 to EW18-2018	114; (111, 117)	1.28; (1.26, 1.31)	EW05-2018; (EW05-2018, EW06-2018)	0.9968	61.50%	114.7	0.99
Ondo state	4.7	Weibull	from EW48-2017 to EW18-2018	128; (118, 138)	1.14; (1.13, 1.15)	EW06-2018; (EW05-2018, EW06-2018)	0.9947	36.40%	126.3	0.003
Ondo state	4.7	Model average	from EW48-2017 to EW18-2018	114; (111, 117)	1.28; (1.26, 1.31)	EW05-2018; (EW05-2018, EW06-2018)	NA	NA	NA	NA
Ebonyi state	2.9	Baseline: AR(2)	from EW45-2017 to EW18-2018	NA	NA	NA	0.9937	0.00%	117.8	NA
Ebonyi state	2.9	Richards	from EW45-2017 to EW18-2018	–	–	–	–	–	–	–
Ebonyi state	2.9	Logistic	from EW45-2017 to EW18-2018	63; (62, 64)	1.55; (1.51, 1.58)	EW08-2018; (EW08-2018, EW08-2018)	0.9982	0.708	90	0.999
Ebonyi state	2.9	Gompertz	from EW45-2017 to EW18-2018	–	–	–	–	–	–	–
Ebonyi state	2.9	Weibull	from EW45-2017 to EW18-2018	66; (64, 69)	1.09; (1.09, 1.09)	EW07-2018; (EW07-2018, EW07-2018)	0.997	52.60%	104.6	0.001
Ebonyi state	2.9	Model average	from EW45-2017 to EW18-2018	63; (62, 64)	1.55; (1.51, 1.58)	EW08-2018; (EW08-2018, EW08-2018)	NA	NA	NA	NA
Bauchi state	6.5	Baseline: AR(2)	from EW49-2017 to EW16-2018	NA	NA	NA	0.9734	0.00%	50.2	NA
Bauchi state	6.5	Richards	from EW49-2017 to EW16-2018	12; (11, 13)	1.34; (1.24, 1.44)	EW12-2018; (EW11-2018, EW13-2018)	0.9936	76.00%	26.5	0.483
Bauchi state	6.5	Logistic	from EW49-2017 to EW16-2018	13; (12, 14)	1.43; (1.37, 1.48)	EW12-2018; (EW11-2018, EW12-2018)	0.993	73.70%	26.4	0.515
Bauchi state	6.5	Gompertz	from EW49-2017 to EW16-2018	–	–	–	–	–	–	–
Bauchi state	6.5	Weibull	from EW49-2017 to EW16-2018	17; (12, 21)	1.09; (1.08, 1.09)	EW12-2018; (EW10-2018, EW13-2018)	0.9889	58.40%	37.5	0.002
Bauchi state	6.5	Model average	from EW49-2017 to EW16-2018	13; (12, 14)	1.38; (1.31, 1.46)	EW12-2018; (EW11-2018, EW13-2018)	NA	NA	NA	NA
Plateau state	4.2	Baseline: AR(2)	from EW48-2017 to EW16-2018	NA	NA	NA	0.9576	0.00%	45.1	NA
Plateau state	4.2	Richards	from EW48-2017 to EW16-2018	–	–	–	–	–	–	–
Plateau state	4.2	Logistic	from EW48-2017 to EW16-2018	9; ( 8, 10)	1.40; (1.32, 1.47)	EW12-2018; (EW11-2018, EW13-2018)	0.9847	63.90%	26.4	0.301
Plateau state	4.2	Gompertz	from EW48-2017 to EW16-2018	–	–	–	–	–	–	–
Plateau state	4.2	Weibull	from EW48-2017 to EW16-2018	16; ( 6, 25)	1.07; (1.05, 1.09)	EW14-2018; (EW09-2018, EW19-2018)	0.9872	69.70%	24.7	0.699
Plateau state	4.2	Model average	from EW48-2017 to EW16-2018	14; (7, 20)	1.17; (1.13, 1.21)	EW13-2018; (EW10-2018, EW17-2018)	NA	NA	NA	NA
Edo state	4.3	Baseline: AR(2)	from EW46-2018 to EW13-2019	NA	NA	NA	0.9919	0.00%	119	NA
Edo state	4.3	Richards	from EW46-2018 to EW13-2019	–	–	–	–	–	–	–
Edo state	4.3	Logistic	from EW46-2018 to EW13-2019	210; (194, 225)	1.37; (1.32, 1.42)	EW07-2019; (EW06-2019, EW07-2019)	0.9907	−14.60%	127.3	excluded
Edo state	4.3	Gompertz	from EW46-2018 to EW13-2019	232; (207, 257)	1.23; (1.19, 1.27)	EW06-2019; (EW05-2019, EW06-2019)	0.994	25.70%	120.6	excluded
Edo state	4.3	Weibull	from EW46-2018 to EW13-2019	307; (230, 384)	1.09; (1.08, 1.10)	EW07-2019; (EW05-2019, EW09-2019)	0.9947	34.40%	118.1	1
Edo state	4.3	Model average	from EW46-2018 to EW13-2019	307; (230, 384)	1.09; (1.08, 1.10)	EW07-2019; (EW05-2019, EW09-2019)	NA	NA	NA	NA
Ondo state	4.8	Baseline: AR(2)	from EW44-2018 to EW13-2019	NA	NA	NA	0.9941	0.00%	104.5	NA
Ondo state	4.8	Richards	from EW44-2018 to EW13-2019	165; (155, 176)	1.31; (1.21, 1.41)	EW06-2019; (EW05-2019, EW07-2019)	0.9927	−24.00%	115.2	excluded
Ondo state	4.8	Logistic	from EW44-2018 to EW13-2019	170; (161, 179)	1.37; (1.33, 1.42)	EW05-2019; (EW05-2019, EW06-2019)	0.9922	−32.40%	114.7	excluded
Ondo state	4.8	Gompertz	from EW44-2018 to EW13-2019	176; (162, 190)	1.28; (1.23, 1.34)	EW04-2019; (EW04-2019, EW05-2019)	0.991	−53.30%	119.9	excluded
Ondo state	4.8	Weibull	from EW44-2018 to EW13-2019	193; (165, 221)	1.09; (1.09, 1.10)	EW05-2019; (EW04-2019, EW06-2019)	0.9895	−78.00%	123.2	excluded
Ondo state	4.8	Model average	from EW44-2018 to EW13-2019	NA	NA	NA	NA	NA	NA	NA
Ebonyi state	3	Baseline: AR(2)	from EW48-2018 to EW13-2019	NA	NA	NA	0.9692	0.00%	89.5	NA
Ebonyi state	3	Richards	from EW48-2018 to EW13-2019	–	–	–	–	–	–	–
Ebonyi state	3	Logistic	from EW48-2018 to EW13-2019	41; (36, 46)	1.43; (1.32, 1.54)	EW07-2019; (EW07-2019, EW08-2019)	0.9749	18.40%	91.2	excluded
Ebonyi state	3	Gompertz	from EW48-2018 to EW13-2019	–	–	–	–	–	–	–
Ebonyi state	3	Weibull	from EW48-2018 to EW13-2019	59; (35, 83)	1.10; (1.08, 1.13)	EW08-2019; (EW05-2019, EW10-2019)	0.9853	52.40%	83.5	1
Ebonyi state	3	Model average	from EW48-2018 to EW13-2019	59; (35, 83)	1.10; (1.08, 1.13)	EW08-2019; (EW05-2019, EW10-2019)	NA	NA	NA	NA
Bauchi state	6.7	Baseline: AR(2)	from EW48-2018 to EW13-2019	NA	NA	NA	0.9657	0.00%	74	NA
Bauchi state	6.7	Richards	from EW48-2018 to EW13-2019	–	–	–	–	–	–	–
Bauchi state	6.7	Logistic	from EW48-2018 to EW13-2019	49; (44, 53)	1.34; (1.27, 1.40)	EW05-2019; (EW04-2019, EW06-2019)	0.979	38.80%	69.7	0.002
Bauchi state	6.7	Gompertz	from EW48-2018 to EW13-2019	–	–	–	–	–	–	–
Bauchi state	6.7	Weibull	from EW48-2018 to EW13-2019	137; ( 8, 266)	1.06; (1.03, 1.09)	EW15-2019; (EW02-2019, EW41-2019)	0.9907	72.90%	57.1	0.998
Bauchi state	6.7	Model average	from EW48-2018 to EW13-2019	137; (8, 266)	1.06; (1.03, 1.09)	EW15-2019; (EW02-2019, EW40-2019)	NA	NA	NA	NA
Plateau state	4.3	Baseline: AR(2)	from EW48-2018 to EW13-2019	NA	NA	NA	0.9679	0.00%	72.2	NA
Plateau state	4.3	Richards	from EW48-2018 to EW13-2019	35; (33, 37)	1.37; (1.23, 1.49)	EW06-2019; (EW04-2019, EW07-2019)	0.9875	61.10%	61.6	0.28
Plateau state	4.3	Logistic	from EW48-2018 to EW13-2019	36; (34, 38)	1.52; (1.43, 1.60)	EW05-2019; (EW04-2019, EW05-2019)	0.9851	53.70%	62.7	0.161
Plateau state	4.3	Gompertz	from EW48-2018 to EW13-2019	36; (34, 37)	1.50; (1.38, 1.61)	EW05-2019; (EW04-2019, EW05-2019)	0.9874	60.60%	61.8	0.253
Plateau state	4.3	Weibull	from EW48-2018 to EW13-2019	37; (34, 39)	1.14; (1.13, 1.15)	EW05-2019; (EW04-2019, EW05-2019)	0.9876	61.40%	61.4	0.306
Plateau state	4.3	Model average	from EW48-2018 to EW13-2019	36; (34, 38)	1.35; (1.27, 1.43)	EW05-2019; (EW04-2019, EW06-2019)	NA	NA	NA	NA

The ‘improvement’ is the partial *R*-squared. The ‘weight’ is the AIC-weight of the selected model, which is used for calculating the model-averaged estimates. The ‘NA’ means a summary term that is not applicable to a certain model. The notation ‘–’ means that the model cannot achieve a converging fitting outcome. The model-averaged estimates in each region are highlighted in grey.

Many previous studies adopted the instantaneous reproduction number, commonly denoted by *R*_*t*_, which can be estimated by a renewable equation, to quantify the transmissibility of infectious diseases [21, 23, 24, 37, 38]. The factors that affect the changing dynamics of *R*_*t*_ include

- the depletion of the susceptible population [32] or decrease in the exposure to infectious sources,
- the change, usually it is the improvement, in the unmeasurable disease control efforts, e.g. contract tracing, travel restriction, school closure, etc., [39–42] and local awareness of the epidemic [33], and
- the natural features of the pathogen, e.g. its original infectivity and other interepidemic factors [32, 33, 35].

In this work, we choose to use the average reproduction number (*R*) rather than *R*_*t*_, as the measurement of the LASV transmissibility. The estimated *R* summarises the LASV transmissibility over the whole period of an epidemic. The reasons why we prefer *R* rather than *R*_*t*_ are as follows. First, the temporal changes of the susceptible population or decrease in the exposure to infectious sources are removed from the *R* estimates due to the nature of the growth models. Second, the changes of the susceptible population and/or disease awareness or control measures and the effect of the rainfall cannot be disentangled in the time-varying reproduction number, *R*_*t*_, the average reproduction number (*R*) adopted is a better proxy to explore the association between LF infectivity and rainfall. With respect to point (iii) and other heterogeneities of epidemics in different regions, we account for this issue by including the ‘region' dummy variables in the LMER model in Eqn (3). These dummy variables serve as random effects to offset the regional heterogeneities of LF epidemics. Therefore, we can then quantify a general effect, i.e. the *β* in Eqn (3), of the lagged rainfall on the LASV *R* estimate among different Nigerian places.

**Fig. 5.**
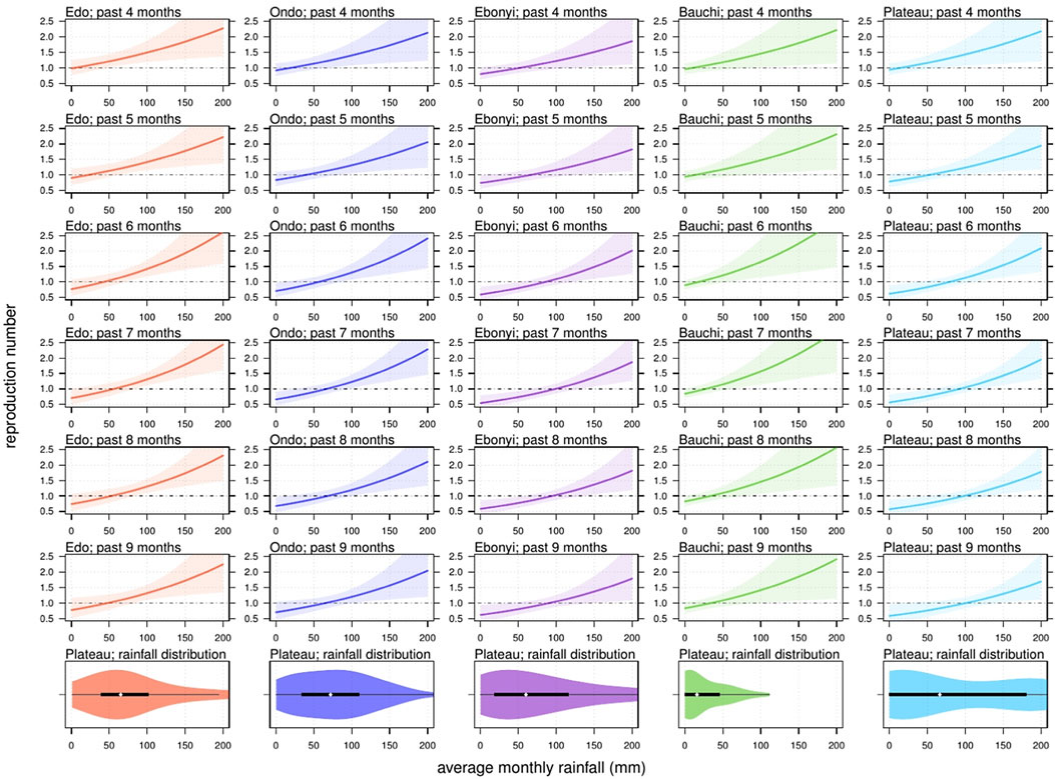
The relationship between state rainfall and Lassa fever (LF) transmissibility, i.e. the reproduction number (*R*), in five different states with different time lags (*t*). The reproduction number of 1.0 is highlighted by the back dashed line. The panels at the bottom are the violin plots and show the distribution of rainfall in each state. The black rectangles represent the 25% and 75% quantiles and the white dot is the median.

The association between total rainfall in a state and the LASV transmissibility (*R*) is modelled and quantified by the LMER model. In Figure 5, we find a positive relation between rainfall and *R*. The estimated changing rate in *R* under a one-unit (mm) increase in the average monthly rainfall is summarised with different cumulative lag terms from 4 to 9 months (the *t* in Eqn (3)). The range of lag in the rainfall from 4 to 9 months had previously been explained by the time interval between the peak of the rainfall and the peak of the rodent population [7]. The estimates of the rainfall-associated changing rate in *R* with different lag terms were summarised in Table 2. We report the most significant (i.e. with the lowest *P*-value) regression estimates that appear with a cumulative lag of 7 months. The habitats of the LASV reservoir, i.e. rodents, include irrigated and flooded agricultural lands that are commonly found in and around African villages [6]. The 7-month lag also coincides with the period between the dry and rainy seasons [43]. The association between rodent population dynamics and rainfall levels has been demonstrated in a number of previous studies [6–10]. Hence, we consider the 7-month lagged estimation as our main results. Namely, a one-unit (mm) increase in the average monthly rainfall over the past 7 months is likely to cause a 0.62% (95% CI 0.20%–1.05%) rise in the *R* of the LF epidemic. We also remark that this ‘one-unit (mm) increase in the average monthly rainfall over the past 7 months’ is equivalent to ‘7-unit (mm) increase in the total rainfall over the past 7 months’. The present finding of the impact of lagged rainfall on LF epidemics suggests that the knowledge of such weather-driven epidemics could be gained by referring to past rainfall levels. For instance, if a relatively high amount of rainfall occurs, local measures, such as rodent population control, could be effective to reduce the LF risk. This speculation could also be verified by examining the rodent population data of the Nigerian regions included in this work. The findings in this work are of public health interest and are helpful for policymakers in LF prevention and control.

**Table 2.** The summary table of the LMER model estimates. The ‘cum. lag’ is the lag term for the cumulative effect of the rainfall. The ‘change rate’ in is the percentage change in *R* for per unit (mm) increase in the average monthly rainfall

Cum. lag	Change rate (%) (95% CI)	*P*-value	*R*-squared
4	0.42 (0.02–0.82)	0.039	0.36
5	0.45 (0.04–0.87)	0.034	0.37
6	0.62 (0.15–1.08)	0.012	0.44
7	0.62 (0.20–1.05)	0.006	0.47
8	0.57 (0.12–1.02)	0.016	0.42
9	0.45 (0.36–1.06)	0.044	0.36

On the one hand, our findings suggest the existence of an association between rainfall and LASV transmissibility, which could be affected by the population dynamics of rodents [13]. On the other hand, the positive relation between rainfall and *R* indicates that rainfall, particularly in states with a high LF risk, can be translated as a warning signal for LF epidemics. The modelling framework in this study should be easily extended to other infectious diseases.

### Limitation

Our work contains limitations. As in some African countries, the weather data are available only from a limited number of observatory stations and thus it is not sufficient to capture more accurate spatial variability. In this work, instead of exploring the spatial differences in the associations between rainfall and LF epidemic, we relaxed the setting and studied a general relationship. We qualified the general rainfall-associated changing rate of *R* in Nigeria. For the transmissibility estimation, our growth modelling framework can provide the estimates of *R*, but not the basic reproduction number commonly denoted as *R*_0_. However, according to the theoretical epidemiology [22, 27, 35, 44, 45], the *R*_0_ can be determined by *R*_0_ = *R*/*S*, where *S* denotes the population susceptibility. Although *S* is not involved in our modelling framework, the information of *S* could be acquired from local serological surveillances. The existing literature reported 21.3% seroprevalence among Nigerian humans by the enzyme-linked immunosorbent assay (ELISA) [46]. Hence, the *R*_0_ can be calculated as 1.63 by using *S* = 1–21.3% = 0.787 and the *R* = 1.28 as the average of the 2016–18 LF epidemics. This was a data-driven modelling study, and we quantified the effect of rainfall as a weather-driven force of *R* based on previous ecological and epidemiological evidences [7, 43]. Since the transmission of LASV mainly relies on the rodent population, the factors including seasonality, agricultural land-using, subtropical or tropical forest coverage that could impact rodent ecology should be relevant and helpful in the analysis. However, due to availability of data, the agricultural land-using factors, e.g. pastureland, irrigated land, flooded agricultural land usage and forest coverage were absent in our analysis, which should be studied in the future if they become available.

## Conclusions

The LF epidemic reproduction numbers (*R*) of the whole of Nigeria in 2017–18 (*R* = 1.33 with 95% CI 1.29–1.37) and 2018–19 (*R* ranged from 1.08 to 1.36) are significantly higher than in 2016–17 (*R* = 1.23 with 95% CI 1.22–1.24). There is significant spatial heterogeneity in the LF epidemics of different Nigerian regions. We report clear evidence of rainfall impacts on LF epidemics in Nigeria and quantify this impact. A one-unit (mm) increase in the average monthly rainfall over the past 7 months could cause a 0.62% (95% CI 0.20%–1.05%) rise in the *R*. The state rainfall information has potential to be utilised as a warning signal for LF epidemics.

## Data

All data used for analysis are freely available via online public domains [12, 15].
